# Predictors of Stepping Up to Higher Level of Care Among Eating Disorder Patients in a Partial Hospitalization Program

**DOI:** 10.3389/fpsyg.2021.667868

**Published:** 2021-07-21

**Authors:** Courtney C. Simpson, Terra L. Towne, Anna M. Karam, Joseph M. Donahue, Carly F. Hadjeasgari, Roxanne Rockwell, Walter H. Kaye

**Affiliations:** Department of Psychiatry, University of California, San Diego, San Diego, CA, United States

**Keywords:** partial hospitalization, higher level of care, eating disorder, predictor, residential, inpatient

## Abstract

Partial hospitalization programming (PHP) is a treatment option available for individuals with eating disorders (ED) who have made insufficient progress in outpatient settings or are behaviorally or medically unstable. Research demonstrates that this level of care yields efficacy for the majority of patients. However, not all patients achieve recovery in PHP and later admit to a higher level of care (HLOC) including residential treatment or inpatient hospitalization. Although PHP is an increasingly common treatment choice for ED, research concerning outcome predictors in outpatient, stepped levels of care remains limited. Thus, the current study sought to identify the predictors of patients first admitted to PHP that later enter residential or inpatient treatment. Participants were 788 patients (after exclusions) enrolled in adolescent or adult partial hospitalization programs in a specialized ED clinic. When compared to patients who maintained treatment in PHP, a significantly greater proportion of patients who discharged to a HLOC had previously received ED residential treatment. Moreover, patients who discharged to a HLOC were diagnosed with a comorbid anxiety disorder and reported greater anxious and depressive symptomatology. A logistic regression model predicting discharge from PHP to a HLOC was significant, and lower body mass index (BMI) was a significant predictor of necessitating a HLOC. Supplemental programming in partial hospitalization settings might benefit individuals with previous ED residential treatment experience, higher levels of anxiety and depression, and lower BMIs. Specialized intervention for these cases is both practically and economically advantageous, as it might reduce the risk of rehospitalization and at-risk patients needing to step up to a HLOC.

## Introduction

Eating disorders (EDs) are difficult to treat with high non-response, dropout, and relapse rates (Fassino et al., 2009; Keel and Brown, 2010; Abbate-Daga et al., 2013). As such, there is a need to better understand the factors that impact both treatment response and long-term outcomes. Identifying predictors of these factors is necessary to improve therapy protocols, inform treatment planning, and identify patients at risk for unfavorable prognoses (Keel and Brown, 2010; Vall and Wade, 2015). An important consideration when examining treatment predictors is the level of care the patient is receiving.

Treatment at partial hospitalization programs (PHP) is most often intended for individuals who have made insufficient progress in outpatient ED treatment or have ED-related behavioral or medical instability that requires regular monitoring. Research has demonstrated that the PHP level of care is effective for many patients (Brown et al., 2018; Reilly et al., 2020). PHPs represent attractive treatment choices to patients, clinicians, and insurance providers, as they demonstrate improved cost-effectiveness and comparable outcomes to inpatient and residential treatment (Anderson et al., 2017). However, not all patients achieve recovery in PHP treatment settings. Thus, some of these individuals later seek admission to a higher level of care (HLOC), including inpatient hospitalization and residential treatment (Abbate-Daga et al., 2015). Although PHP settings are an increasingly common treatment choice for individuals with EDs, research concerning outcome predictors in these settings remains limited. In a recent meta-analysis of outcome predictors, 67% of included studies were from randomized controlled trials and reflected specific treatment settings: 51.5% inpatient, 32.5% outpatient, 1.6% PHP and 0.8% residential settings (Vall and Wade, 2015). As randomized control trial findings might not reflect outcomes in more naturalistic settings, such as outpatient or stepped-level of care settings (e.g., PHP), additional research is warranted to replicate results and identify other variables that might impact ED treatment outcomes in these environments (Vall and Wade, 2015; Walker et al., 2020).

Previous research, not necessarily pertaining to PHP settings as mentioned above, has identified numerous baseline variables that predict outcomes and mediators that help explain favorable treatment response, including: higher body mass index (BMI), fewer binge/purge episodes, increased motivation to recover, lower shape/weight concern, fewer comorbidities, and better interpersonal functioning (Vall and Wade, 2015; Linardon et al., 2016). Several studies have also evaluated whether age is a predictor of outcome, with inconsistent results. For example, some studies have found younger age predicts more favorable outcome in outpatient settings (e.g., Agras et al., 2014), while others have demonstrated the opposite pattern of older age predicting better outcome (Grilo et al., 2012), or found no association between age and treatment outcome (e.g., Lammers et al., 2015). Temperament represents a relatively new area of focus associated with ED treatment outcome (Kaye et al., 2015); five-year follow-up from outpatient treatment suggests that temperamental traits such as low novelty seeking, high harm avoidance, and high reward dependence predict clinical improvement in ED symptoms (Segura-García et al., 2013). Similarly, emotion dysregulation has recently been identified as a critical mechanism in the development and maintenance of EDs (Lavender, 2015) with greater emotion regulation skills predicting favorable ED outpatient treatment outcomes (MacDonald et al., 2017).

Although identifying factors that predict favorable treatment outcomes is essential, it is equally—if not more important—to identify variables that predict poor prognosis. A systematic and meta-analytic review by Vall and Wade (2015), looking mostly at data from inpatient and outpatient settings, demonstrated that higher eating pathology at baseline predicted worse outcomes. Another systematic review evidenced a consistent link between anxiety and depression and worse ED treatment outcomes (Berkman et al., 2007). Smith et al. (2018) identified baseline general anxiety, as well as social anxiety, as significant predictors of poor end of treatment ED psychopathology in residential settings. Accurso et al. (2016) found that higher depression scores predicted more ED psychopathology at short-term follow-up from outpatient treatment, and two studies examining outpatient outcomes at 12-month follow-up found that higher depression scores and the presence of major depression were associated with more episodes of binge eating and purging (Fahy and Russell, 1993; Bulik et al., 1998). Further, research has consistently revealed that longer duration of illness and lower body mass index (BMI) at baseline are poor prognostic factors (Howard et al., 1999; Reas et al., 2000; Pinter et al., 2004).

Although research has identified general predictors for unfavorable treatment outcomes, a critical yet understudied subset of patients with poor outcomes are those that enter PHP for an ED but later require a HLOC due to needing more support and intensive treatment. As treatment in PHP levels are an increasingly common and attractive choice for ED patients, it is crucial to understand which types of patients will have favorable outcomes in these settings. Moreover, it is important to identify patients early on in treatment who often need or could benefit from a HLOC. Further, elucidating PHP patient characteristics that necessitate a HLOC is advantageous in identifying and implementing supplemental programming in PHP settings that might benefit individuals experiencing severe eating pathology and potentially prevent having to step up to a HLOC (Fewell et al., 2017).

As noted, there is a lack of literature examining outcome predictors at the PHP level, and there is limited research that evaluates the clinical indicators that predict unfavorable outcomes. As such, the purpose of the current study was to better characterize PHP patients who require a HLOC and identify predictors of those who discharged to a HLOC (i.e., residential or inpatient) after an initial PHP admission. Specifically, we aimed to characterize patients who require a HLOC by examining their demographic and baseline clinical characteristics and differences on these variables between patients who discharged to a HLOC and patients appropriate for PHP. Although the current study was exploratory in nature, it also aimed to assess previously identified predictors of treatment outcome (i.e., anxiety, depression, eating pathology, duration of illness, age, BMI) as predictors of patients requiring a HLOC.

## Methods

### Participants and Procedure

Nine-hundred sixty-three patients who admitted to PHP participated in the present study. Eight were excluded for missing reason for discharge data. Eighty of the 955 remaining participants readmitted to PHP at a later date, and only data from their initial admissions were used in the present study. This decision was intended to identify the maximum number of participants who discharged to a HLOC while also differentiating patients who discharged to a HLOC from patients who were appropriate for continued PHP (i.e., by reducing the chances that patients who enrolled in PHP and discharged to a HLOC before returning to PHP would be included in the “appropriate for continued PHP group” and compared to those who discharged to a HLOC). However, because treatment history data were not collected until July 2016, the 501 participants who participated in the study prior to this date may have attended the current study's PHP in the past. Six participants who re-admitted to PHP at a later date were excluded from the present study due to having no available data from their initial admission. Participants whose only involvement with the current study's program prior to PHP admission was the 5-day Intensive Family Treatment program (*n* = 3) were included in the present study.

Of the 949 total participants, 728 were classified as appropriate for PHP, as evidenced by reasons for discharge indicating a completed treatment course or need for further PHP. Specifically, 631 discharged appropriately, 46 discharged due to insurance reasons, 43 discharged for personal reasons or to return to their college, work, or non-local residence, and 8 discharged to a different PHP. Participants who discharged from PHP against medical advice (*n* = 132) or as a result of failing a therapeutic contract (*n* = 29) were excluded from analyses due to the nuanced and heterogeneous nature of these discharges that cannot be adequately captured by a binary variable. For example, some participants who discharged against medical advice or failed a therapeutic contract may have been appropriate for further PHP, while others may have discharged upon a higher level of care being recommended or presented as a contingency of failing a therapeutic contract. Following admission to PHP, sixty patients discharged to a higher level of care, with 43 discharging to a residential treatment center, 13 discharging to inpatient hospitalization for imminent suicidality or suicide attempts, and four discharging to inpatient hospitalization for acute weight loss and/or medical instability.

Admission criteria for PHP were in line with the American Psychiatric Association's medical, psychiatric, and behavioral criteria guidelines for the treatment of EDs (Yager et al., 2014). Informed consent was obtained from all participants, and an Institutional Review Board approved all study procedures. All patients who admitted to these programs and who voluntarily consented to research involvement were included in the current study.

### Brief Program Overview

The ED PHP where the study took place includes a multidisciplinary team (including a licensed therapist or psychologist, psychiatrist or nurse practitioner, dietitian, and nursing staff) that provides regular individual, family, and group therapy, medication management, meal support, and dietary consultation. Upon admission to PHP, patients attend treatment for 10 hours per day, 6 days per week. As symptoms improve, patients step down to intensive outpatient programming before discharging to regular outpatient care. Adult programming utilized a dialectical behavior therapy (DBT) model [see Brown et al. (2018) for more details] whereas adolescent programming used a family-based therapy (FBT)-DBT approach [see Reilly et al. (2020) for more details].

Patient characteristics and demographic data were collected only from patients who entered at the PHP-level of care, and included: patients' age, sex, gender identity, race, ethnicity, ED diagnosis, duration of illness, and BMI, as well as diagnoses of mood, anxiety, and alcohol and substance use disorders. The question assessing gender identity was added to the study at a later date and may not fully represent the gender identities of the full sample; prior to the addition of this variable, the extent to which participants reported their gender as sex is unknown. As the data used in this study have been collected over many years, the methods used to determine ED and comorbid diagnoses have varied. Some patients' diagnoses were determined by staff psychiatrists and nurse practitioners at admission, while others were diagnosed using structured clinical interviews such as the *Structured Clinical Interview for DSM-5* (First et al., 2015; SCID) or the *Mini International Neuropsychiatric Interview* (Sheehan et al., 1998; MINI).

Participants mostly identified their sex as female (91.6% female).The most commonly identified gender identities were female (87.2%) and male (8.8%), though a small number of patients identified as gender-non-conforming (3.3%) and different identity (0.7%). Patients were a mean age of 20.87 (SD = 8.35), and just over half (52.6%) were adults. In terms of race and ethnicity, 73.3% identified as Caucasian, 6.5% as Asian, 1.5% as Black, 0.9% identified as either Native Hawaiian/Pacific Islander or Native American/Alaskan Native, 17.8% identified as other, and 18.4% identified as Hispanic. The ED diagnostic breakdown of the sample is as follows: 49.5% Anorexia Nervosa Restricting type (AN-R), 12.7% Anorexia Nervosa Binge-eating/Purge type (AN-BP), 21.8% Bulimia Nervosa (BN), 2.2% Binge Eating Disorder (BED), 5.8% Avoidant and Restrictive Food Intake Disorder (ARFID), 8.9% Other Specified Feeding and Eating Disorder (OSFED), and 0.1% Unspecified Feeding and Eating Disorder (USFED).

Average duration of ED was 6.03 years (SD = 7.51) and mean admit BMI was 20.19 kg/m^2^ (SD = 4.76). In terms of current comorbidities, 54.3% of the sample had a mood disorder, 53.8% had an anxiety disorder, 5.7% had alcohol use disorder, and 9.0% had substance use disorder.

### Measures

Previous HLOC ED treatment was assessed using a single item question which asked, “Have you previously been in treatment for an eating disorder?” Response options included inpatient, residential, PHP, and outpatient. BMI (kg/m^2^) was calculated using height and weight measured in the clinic upon admission.

#### Eating Disorder Examination Questionnaire

The eating disorder examination questionnaire (EDE-Q; Fairburn and Beglin, 1994) is a 31-item self-report questionnaire that assesses the severity of ED psychopathology over the past 28 days. This study used the EDE-Q global score, which averages across symptom subscales (e.g., restraint, weight concern, eating concern, shape concern) to provide a general indication of cognitive eating pathology. In our sample, the EDE-Q global subscale showed excellent internal consistency across time (α = 0.97).

#### The State and Trait Anxiety Inventory

The State–Trait Anxiety Inventory—Trait subscale (STAI-T; Spielberger et al., 1970) is a 20-item self-report measure that assesses trait anxiety. The STAI-T subscale demonstrated good internal consistency in the current study (α = 0.95).

#### Difficulties in Emotion Regulation Scale

The Difficulties in Emotion Regulation Scale (DERS; Gratz and Roemer, 2004) is a 36-item scale used to measure emotion regulation difficulties. The current study used the DERS total score, with higher scores indicating greater difficulty with emotion regulation. Previous research has indicated this measure has sound psychometric properties in samples of both adults and adolescents (Gratz and Roemer, 2004; Neumann et al., 2010). Internal consistency in the present study was good (DERS total α = 0.96).

#### The Sensitivity to Punishment/Sensitivity to Reward Questionnaire

The Sensitivity to Punishment/Sensitivity to Reward Questionnaire (SPSRQ; Torrubia et al., 2001; Franken and Muris, 2006) is a self-reported instrument that includes 48 yes/no questions. This measure is divided into two subscales: Sensitivity to Reward (SR; α = 0.81) and Sensitivity to Punishment (SP; α = 0.89).

#### The Beck Depression Inventory

The Beck Depression Inventory (BDI-II) is a 21-item, self-report rating inventory that measures characteristic attitudes and symptoms of depression (Beck et al., 1996). Symptoms during the past 2 weeks using a variable rating scale (i.e., 19 items use a 4-point scale, two items use a 7-point scale). Internal consistency in the present study was good (BDI total α = 0.93).

### Data Analysis

Means, standard deviations, and frequencies were calculated to describe both patients who admitted to a HLOC following PHP admission and patients who continued to be treated in PHP. *T*-tests and chi-square tests were used to detect group differences on clinical and demographic variables and examine patterns of missing data. Bonferroni corrections were applied to control for multiple comparisons.

Firth logistic regression with penalized maximum likelihood estimation was used to examine predictors of admitting to a HLOC following PHP admission. This analysis was selected to mitigate imbalance and separation issues inherent in predicting rare events that comprise a small proportion of the total sample. Because this was the first study to examine predictors of needing a HLOC among PHP patients, we selected predictors both based on established correlates and predictors of poor treatment prognosis and on group differences in the present study between those who stepped up to HLOC and those who were appropriate for PHP. Of the five variables that differed between the two treatment groups, three were included in logistic regression models, while two were excluded due to being highly correlated (ρ > 0.80) with other variables in the model. Lastly, a binary program term (adolescent vs. adult program) was included as a predictor in the models.

As such, individuals who did and did not necessitate admission to a HLOC following PHP admission were regressed upon ED diagnosis, duration of illness, BMI, eating pathology (i.e., EDE-Q global score), trait anxiety (i.e., STAI trait subscale), depression (i.e., BDI total score), and eating disorder program (i.e., adolescent or adult program). To ensure an adequate number of participants per cell, the ED diagnosis variable was condensed into three categories (i.e., AN, BN, OSFED); for the purposes of this analysis, the OSFED category contained patients with DSM-V diagnoses of ARFID, BED, OSFED, and USFED. In the first model, the higher level of care group consisted of all patients who admitted to a HLOC following PHP admission; patients who admitted to the hospital due to suicidality and/or suicide attempts were excluded from the second model.

## Results

Patients with missing reasons for discharge data (*n* = 8) were significantly younger [*t*_(796)_ = 5.98, *p* < 0.001] and had a shorter duration of illness [*t*_(777)_ = −6.55, *p* < 0.001] when compared to patients whose reasons for discharge were documented. There were no group differences on missing/non-missing reasons for discharge data on any of the following continuous and categorical variables: eating disorder psychopathology, age of onset, admit or discharge BMI, trait anxiety, depressive symptoms, emotion dysregulation, sensitivity to punishment, race, ethnicity, or diagnoses of comorbid depressive, anxiety, alcohol use, and substance user disorders (*p*s > 0.05). Individuals with missing data on anxiety [χ^2^_(1, N = 793)_ = 6.33, *p* = 0.02] and depressive disorders [χ^2^_(1, N = 793)_ = 5.66, *p* = 0.02] were more likely to discharge to a higher level of care, while missing data on all other baseline variables were not related to reason for discharge (*p*s > 0.05).

When compared to patients who were appropriate for continued PHP, a significantly greater proportion of patients who discharged to a HLOC had previously received ED treatment at residential treatment centers (ϕ_c_ = 0.21) and were diagnosed with comorbid anxiety disorders (ϕ_c_ = 01). Moreover, patients who discharged to a HLOC had a lower BMI (*d* = 0.58) and reported greater anxious (*d* = 0.71) and depressive (*d* = 0.67) symptomatology than those who were appropriate for continued PHP. See Table 1 for details.

**Table 1:** Patient and clinical characteristics at PHP admission.

	**Stepped up to HLOC**	**Appropriate for PHP**	***t* or χ ^**2**^ value**	***p*-value**
Age, mean years (±SD)	19.15 (6.33)	21.01 (8.48)	2.11	0.04
PHP program attended			0.49	0.47
Adolescent (< 18 years)	31 (52.67%)	345 (47.07%)		
Adult (18+ years)	29 (48.33%)	388 (52.93%)		
Sex, *n* (%)			-	-
Female	74 (100%)	677 (92.11%)		
Male	0 (0%)	58 (7.89%)		
Race, *n* (%)			0.1	0.75
Caucasian	45 (75%)	531 (73.14%)		
Asian	4 (6.67%)	47 (6.47%)		
African American	1 (1.67%)	11 (1.52%)		
Native American/Alaskan Native	0 (0%)	3 (0.41%)		
Native Hawaiian/Pacific Islander	0 (0%)	4 (0.55%)		
Other racial background	10 (16.67%)	130 (17.91%)		
Ethnicity, *n* (%)			0.01	0.91
Hispanic/Latino	11 (18.97%)	132 (18.33%)		
Non-Hispanic/Latino	47 (81.03%)	588 (81.67%)		
Eating disorder diagnosis, *n* (%)			4.28	0.09
AN-R	32 (55.17%)	359 (49.04%)		
AN-BP	12 (20.69%)	88 (12.02%)		
BN	11 (18.97%)	161 (21.99%)		
BED	0 (0%)	17 (2.32%)		
ARFID	1 (1.72%)	45 (6.15%)		
OSFED	2 (3.45%)	61 (8.33%)		
USFED	0 (0%)	1 (0.14%)		
Duration of illness mean years (±SD)	5.56 (6.38)	6.07 (7.59)	0.49	0.57
Mean admit BMI (±SD)	18.67 (3.40)	20.31 (4.83)	−3.47	0.001^*^
Comorbidities, *n* (%)				
Mood disorder	31 (55.36%)	390 (54.17%)	0.03	0.86
Anxiety disorder	40 (71.43%)	377 (52.43%)	7.54	0.006^*^
Alcohol use disorder	2 (3.57%)	42 (5.84%)	-	-
Substance use disorder	8 (14.29%)	62 (8.62%)	2.03	0.15
Treatment history, *n* (%)
Residential	11 (57.89%)	48 (24.37%)	9.81	0.002^*^
Inpatient	9 (47.37%)	89 (45.18%)	0.03	0.85
PHP	9 (35.18%)	40 (20.31%)	1.31	0.25
IOP	9 (47.37%)	53 (26.77%)	3.55	0.06
Outpatient	18 (94.74%)	133 (67.51%)	6.12	0.01
Psychopathology and temperament, *n* (%)
EDEQ global	4.03 (1.55)	3.50 (1.69)	2.56	0.01
State anxiety	63.67 (11.37)	58.49 (12.92)	3.35	0.001^*^
BDI-II score	32.30 (11.97)	26.99 (13.86)	3.03	0.004^*^
DERS score	116.48 (26.00)	111.06 (29.64)	1.42	0.16
Punishment sensitivity mean	15.05 (5.87)	16.56 (6.84)	0.7	0.5

*Percentages were calculated based on available data. Group differences on gender identity, sex, and alcohol use disorder were not assessed due to one or more categories per variable containing < 5 cases. Group differences on eating disorder diagnosis were assessed by grouping the variable into three categories (AN, BN, and all other eating disorders); group differences on race were assessed by grouping the variable into patients identifying as Caucasian and patients identifying as Black, Indigenous, and People of Color (BIPOC). p-values ≤ 0.006 were considered statistically significant*.

*AN-R, Anorexia Nervosa Restricting Type; AN-BP, Anorexia Nervosa Binge Purge Type; BN, Bulimia Nervosa; BED, Binge Eating Disorder; ARFID, Avoidant and Restrictive Food Intake Disorder; OSFED, Other Specified Feeding and Eating Disorder; USFED, Unspecified Feeding and Eating Disorder; PHP, Partial Hospitalization Program; IOP, Intensive Outpatient Program*.

* *p ≤ 0.006*.

When all patients were included in the first logistic regression, the overall model was significant in predicting the dependent variable [i.e., admission to a HLOC following PHP admission; Likelihood Ratio χ^2^_(8)_ = 22.94, *p* = 0.003]. Only BMI significantly predicted discharging to a HLOC. Specifically, for every one unit decrease in BMI, participants were 15% more likely to admit to a HLOC (Table 2). Upon excluding patients who were hospitalized for suicidality and/or suicide attempts following PHP admission, the overall model remained statistically significant [Likelihood Ratio χ^2^_(8)_ = 21.57, *p* = 0.005]. BMI remained the only significant predictor (χ^2^ = 10.91, OR = 0.78, *p* = 0.001), such that every one unit decrease in BMI resulted in a 22% greater likelihood of admitting to a HLOC. This model's restricted log likelihood value (−113.27) was closer to zero than in the original model (−143.17), suggesting model fit is improved when patients who were hospitalized for suicidality and/or suicide attempts were excluded from the analysis.

**Table 2:** Predictors of discharging to a higher level of care following PHP admission.

**Variable**	***B* (SE)**	**χ^2^**	**Exp(*B*)**	***p*-value**
Intercept	−1.26(1.49)	0.66	0.28	0.42
ED diagnosis
AN^a^	-	-	-	-
BN	0.39 (0.49)	0.59	1.47	0.44
OSFED	−0.75 (0.69)	1.31	0.47	0.25
Duration of illness (years)	0.02 (0.02)	0.80	1.02	0.37
BMI	−0.16 (0.07)	6.67	0.85	0.01^*^
EDEQ Global	0.15 (0.14)	1.14	1.16	0.29
Treatment Program (adult or adolescent)	0.13 (0.35)	0.67	1.14	0.73
Trait anxiety	0.01 (0.02)	0.33	1.01	0.57
BDI	0.01 (02)	0.36	1.01	0.55

a *Indicates comparison group for non-binary categorical variables*.

*ED, Eating Disorder; AN, Anorexia Nervosa; BN, Bulimia Nervosa; OSFED, Other Specified Feeding and Eating Disorders; BMI, Body Mass Index; EDEQ, Eating Disorder Examination Questionnaire; BDI, Beck Depression Inventory*.

* *p < 0.05*.

## Discussion

The current study sought to identify predictors of patients first admitted into a PHP level of care who later required a step up to residential or inpatient treatment settings. Findings demonstrate that patients with lower BMIs were more likely to admit to a HLOC from PHP. Data indicate that this finding remains statistically significant when psychiatric hospitalizations for suicidality and/or suicide attempts were excluded. Results highlight the importance of considering BMI when developing treatment plans for newly admitted patients to PHP settings.

Previous research demonstrates that lower BMI at pretreatment is a poor prognostic factor (Fahy and Russell, 1993; Howard et al., 1999; Agras et al., 2000; Pinter et al., 2004; Berkman et al., 2007). One study identified lower BMI at the time of inpatient admission predicts PHP failure and inpatient readmission for patients with anorexia nervosa (Howard et al., 1999). Another study conducted in an inpatient setting highlighted that patients with anorexia nervosa with BMIs below 15 kg/m^2^ were significantly more likely to develop a lower BMI at follow-up (Pinter et al., 2004). Two studies examining bulimia nervosa in outpatient settings identified that lower BMI pretreatment was associated with worse outcome in terms of binge/purge frequency and eating disorder psychopathology at end of treatment and short-term follow-up (Fahy and Russell, 1993; Agras et al., 2000). The current study adds to the previous literature in revealing that transdiagnostic patients with EDs with lower BMIs at admission, specifically in PHP settings, were more at risk for stepping up to HLOC. As such, patients across ED diagnoses with lower BMIs might demonstrate greater need for more comprehensive, targeted interventions, specifically in regards to nutrition, when admitting to PHP settings.

Exploration of group differences revealed that when compared to patients appropriate for continued PHP, patients who discharged to a HLOC were significantly more likely to have previously received ED residential treatment. This highlights the importance of acknowledging and exploring previous treatment history at admission. Certainly, communication between treatment facilities is necessary to supplement success (Anderson et al., 2017). Further, knowledge of previous treatment can inform the implementation of expectations outlined at admission to a PHP. Setting clear behavioral contingences based on previous treatment that reinforce functional rather than dysfunctional behaviors might engender early change (Wisniewski and Ben-Porath, 2015; Ziser et al., 2018). Establishing an explicit treatment contract collaboratively with patients to align with their personal goals might enhance patients' motivation, compliance, and autonomy (Wisniewski and Ben-Porath, 2015). As such, initiating collaborative contingency contracts at the outset of PHP treatment for patients with a history of residential treatment might augment outcomes.

Additionally, group differences demonstrated that patients who discharged to a HLOC were more likely to be diagnosed with comorbid anxiety disorders and endorsed greater anxious and depressive symptomatology.

Previous research consistently demonstrates that higher levels of anxiety and depression are related to poorer prognosis (Berkman et al., 2007; Vall and Wade, 2015). Indeed, neurobiological research indicates that anxiety inhibits motivation to eat, which maintains the cycle of restriction and weight loss (Frank et al., 2019). As such, combining nutritional rehabilitation with specific biological interventions might supplement treatment outcome (Frank et al., 2019).

### Strengths and Limitations

The current study's strengths include a large sample of adolescent and adult patients presenting for treatment with diverse presentations of ED. Although we consider conducting research in a naturalistic PHP setting a strength, treatment outcomes research within routine clinical practice presents unique limitations. First, therapists within the current study's PHP utilized an evidenced-based framework but practiced independently, and treatment fidelity assessments were not administered. Another limitation to the study is the lack of standard diagnostic assessment across the entire sample. Further, the sample included patients from one PHP, limiting generalizability to other PHP facilities. Also related to the generalizability of these findings is that it is unknown how many patients declined research participation, and if or how patients who did not consent to be involved in research differ from those in this study, and whether the findings would hold if they were included. Lastly, the number of patients in this sample that discharged from PHP to a HLOC was small and comprised just under 8% of the total sample. As such, observed power to detect a small effect (odds ratio = 1.2 or 20% increase in likelihood) in a logistic regression model was just 12%. However, this estimate may not accurately reflect the power achieved using firth logistic regression, the analysis selected for this study due to its appropriateness for predicting rare events among unbalanced groups (King and Langche, 2001). Even so, the present study was likely underpowered to detect predictors of discharge to a HLOC as well as nuanced interaction effects that may exist among predictors.

## Conclusion

To our knowledge, this was the first study to explore clinical predictors of patients who require a HLOC after initial admission to a PHP. Findings indicated targeted early interventions in ED PHP settings might benefit patients with lower BMIs. Additionally, current findings suggested that patients exhibiting higher levels of anxiety and depression and/or reporting previous ED residential treatment experience might also benefit from supplemental intervention strategies. Specialized treatment for these cases is both practically and economically advantageous as it might reduce the risk of re-hospitalization and at-risk patients needing to step up to a HLOC.

## Data Availability Statement

The data the support the findings of this study can be made available from the corresponding author upon request.

## Ethics Statement

The studies involving human participants were reviewed and approved by University of California, San Diego. Written informed consent to participate in this study was provided by the participants' legal guardian/next of kin.

## Author Contributions

All authors listed have made a substantial, direct and intellectual contribution to the work, and approved it for publication.

## Conflict of Interest

The authors declare that the research was conducted in the absence of any commercial or financial relationships that could be construed as a potential conflict of interest.
